# Lipid Traits and Risk of Subarachnoid Haemorrhage: A Systematic Review and Mendelian Randomization Study in UK Biobank

**DOI:** 10.1007/s12975-026-01499-6

**Published:** 2026-10-08

**Authors:** Frederick Ewbank, Cameron Pinn, Benjamin Gaastra, William Tapper, Ian Galea, Diederik Bulters

**Affiliations:** 1 Clinical Neurosciences, Clinical & Experimental Sciences, Faculty of Medicine, University of Southampton, Southampton, Hampshire UK; 2 Department of Neurosurgery, University Hospital Southampton NHS Foundation Trust, Tremona Road, Southampton, Hampshire SO16 6YD UK

**Keywords:** Intracranial aneurysm, Subarachnoid haemorrhage, Stroke, Lipids, Cholesterol, Mendelian randomisation

## Abstract

Lipid traits are established risk factors for atherosclerotic disease; however, their relationship with intracranial aneurysms and subarachnoid haemorrhage (SAH) remains unclear. Recent Mendelian randomisation (MR) studies have reported inconsistent findings. This study aimed to review the literature and assess the association between lipid traits and SAH using a one-sample MR analysis accounting for statin use and nonlinearity. We performed a systematic review of MR studies investigating lipid traits and intracranial aneurysms/SAH. A meta-analysis was not undertaken because studies used overlapping datasets. In parallel, we conducted a one-sample MR analysis of 486,603 UK Biobank participants. Genetic instruments for low-density lipoprotein (LDL), high-density lipoprotein (HDL), total cholesterol (TC), and triglycerides (TG) were derived from published genome-wide association studies. Associations with SAH were adjusted for lipid-lowering therapy. Nonlinear relationships were explored using cubic splines and piecewise MR. While eight MR studies met the inclusion criteria, seven relied on the same outcome cohort. Despite this, these studies showed no consistent direction of effect across lipid traits, suggesting that the estimates were unstable. In the UK Biobank, there was no evidence of an association between genetically predicted lipid traits and SAH for LDL (HR 1.06 [0.64–1.73]), HDL (HR 2.57 [0.42–15.74]), TC (HR 1.06 [0.71–1.60]), or TG (HR 1.05 [0.69–1.59). Results were consistent across different MR methods and sensitivity analyses, including adjustment for lipid-lowering therapy. Current evidence does not support a major association between lipid traits and SAH, with the heterogeneity observed across previous MR studies likely reflecting methodological limitations rather than a consistent biological effect. However, small effects and lipid-independent effects of lipid-lowering therapies cannot be excluded.

**Supplementary Information:**

The online version contains supplementary material available at https://doi.org/10.1007/s12975-026-01499-6.

## Introduction

Subarachnoid haemorrhage (SAH) is a rare form of stroke associated with significant morbidity and mortality [1]. The rupture of intracranial aneurysms accounts for the majority of SAH cases and, subsequently, patients with unruptured intracranial aneurysms (UIA) represent a high-risk group. Despite advances in the endovascular and surgical treatment of UIA, there are no prophylactic medical therapies which can be used to prevent rupture. While there have been advances in risk stratification, little is known about the pathophysiology of ruptured intracranial aneurysms (RIA) [2].

Lipids play a key role in atherosclerotic conditions, such as ischaemic heart disease and stroke. Until recently there has been limited and conflicting data regarding the relationship between lipid traits and SAH. Previous studies have highlighted a protective role of statins [3, 4]. However, inflammation is believed to play an important role in the formation and rupture of intracranial aneurysms, and statins, in addition to lowering cholesterol, have also been shown to inhibit inflammatory pathways involved in aneurysm formation [5–7].

Many observational studies investigating the epidemiology of SAH are limited by an inability to exclude correlated biological pathways. In the absence of pleiotropy, Mendelian randomisation (MR) can provide a more robust estimate of causal effect. Recently, several MR studies investigating the association between lipid traits and SAH have been published with some contradictory results [8–15]. However, none of these studies account for statin use, which can lower lipid levels and potentially attenuate SNP–exposure associations while influencing SNP–outcome associations. Additionally, all studies assume a linear relationship between lipid traits and SAH. However, this association may be nonlinear, and difficulties in fitting a linear model could also contribute to the variation observed across previous studies.

Subsequently, the aim of this study was to characterise the genetic association between lipid traits and SAH by performing (1) a systematic review of the published literature and (2) an MR analysis of patients in the UK Biobank that accounts for statin use and considers both nonlinear and linear effects.

The UK Biobank provides a unique opportunity to perform sensitivity analyses accounting for statin use and to investigate whether adjustment for lipid-lowering therapy influences the estimated associations between genetically predicted lipid traits and SAH. In addition, the ability to perform a one-sample MR enables us to perform nonlinear analyses.

## Methods

### Systematic Review

We followed the “Preferred reporting items for systematic reviews and meta-analyses” (PRISMA) statement for reporting this systematic review. Electronic databases including Medline, Embase, and Google Scholar were searched from inception until 26/02/2026. Search terms for MR (“mendelian randomisation” and “mendelian randomization”) were combined with search terms for SAH (“subarachnoid bleed”, “subarachnoid hemorrhage”, “subarachnoid haemorrhage”, “cerebral aneurysm” and “intracranial aneurysm”).

### Study Selection

Following the removal of duplicates, the titles and abstracts of each study were reviewed by the lead author. Full texts were reviewed for all MR studies reporting on lipid traits and SAH. Abstracts, case reports, conference presentations, editorials, reviews, and expert opinions were excluded. Publications were limited to those involving human subjects. Only studies written in the English language were included.

### Data Extraction

All data regarding high-density lipoprotein (HDL), low-density lipoprotein (LDL), total cholesterol (TC) and triglyceride (TG), including reported odds ratios (OR) and confidence intervals (CI), were extracted from the text and figures presented in the published articles.

### Statistical Analysis

Forest plots were used to visually summarise the reported effect estimates from individual studies. No pooled summary estimates were calculated because most studies used overlapping outcome datasets, meaning that the reported effect estimates were not independent. For each analysis, the heterogeneity of the included studies was analysed using standard χ2 tests. The percentage of variation across the studies was quantified using the I^2^ statistic. I^2^ values of 25%, 50%, and 75% corresponded to low, moderate, and high degrees of heterogeneity, respectively. A p value was considered significant at 0.05. Subgroup analyses were performed looking at patients with RIA and UIA.

### UK Biobank Cohort

The UK Biobank is a prospective population-based cohort study of > 500,000 participants from England, Scotland and Wales. Participants aged 40–69 years old were initially recruited between 2006 and 2010. While follow up is ongoing, in this study, participants were followed up from baseline assessment until 31/3/2017. Participants were censored when deaths were reported prior to this date. This study was conducted as part of UK Biobank application ID 49,305. UK Biobank has National Research Ethics Committee Approval 16/NW/0274 and this study had institutional approval (ERGO 45963, University of Southampton). All data is available from the UK Biobank.

### Genetic Instruments

Genotype calling was performed by Affymetrix on two closely related purpose-designed arrays. Approximately 50,000 participants were run on the UK BiLEVE Axiom array and the remaining 450,000 were run on the UK Biobank Axiom array. GRCh37 coordinates were used to define the position of single nucleotide polymorphisms (SNPs). Information regarding quality control, phasing, imputation, population structure and relatedness has previously been published [16]. Genetic instruments were identified from previously published GWAS [17–19]. SNP F statistics were calculated from the SNP–exposure associations estimated within the UK Biobank as F = (β/SE)² (Supplementary Table 1). Additive SNPs strongly associated (F statistic > 10) with exposure were included. Further analyses were performed using SNPs more strongly associated with exposure (F statistic > 20 and > 50).

### Lipids

Serum lipid measurements were obtained from UK Biobank blood biochemistry data and were measured in mmol/L. Total cholesterol was defined using UK Biobank Field 30690, HDL cholesterol using Field 30760, directly measured LDL cholesterol using Field 30780 and triglycerides using Field 30870.

### Subarachnoid Haemorrhage

SAH cases were identified through linked hospital admission data, including Hospital Episode Statistics – Admitted Patient Care (England) (HES APC), Scottish Morbidity Records – General / Acute Inpatient and Day Case Admissions (Scotland) (SMR01) or Patient Episode Database for Wales (PEDW), and death registry data. This included participants with one (or more) ICD 10 codes associated with SAH (Table 1). Participants with a SAH code date prior to baseline assessment were excluded from this analysis. Cases associated with traumatic brain injury prior to or within 28 days of the SAH were excluded on the basis that they may have been incorrectly coded (Supplementary Fig. 1). Sensitivity analyses were performed including cases more likely to be aneurysmal (Table 1).

**Table 1:** ICD10 codes associated with SAH

Variable		ICD10 code	Definition
Aneurysmal subarachnoid haemorrhage	Specified aneurysm location	I600-5	Non- traumatic subarachnoid haemorrhage including ruptured cerebral aneurysm
	No specified aneurysm location	I606, I607, I609	Non- traumatic subarachnoid haemorrhage including ruptured cerebral aneurysm
Non-aneurysmal subarachnoid haemorrhage		I608	Non- traumatic subarachnoid haemorrhage including meningeal haemorrhage and rupture of cerebral arteriovenous malformation
Traumatic brain injury		S061	Traumatic cerebral oedema
		S062	Diffuse traumatic brain injury
		S063	Focal traumatic brain injury
		S064	Epidural haemorrhage
		S065	Traumatic subdural haemorrhage
		S066	Traumatic subarachnoid haemorrhage
		S068	Other specified intracranial injuries
		S069	Unspecified intracranial injuries

### Statins

Medication use was self-reported at baseline and follow-up assessment centre visits (UK Biobank Field 20003). Data on prescription medications was obtained through a verbal interview by a trained nurse. Only treatments taken regularly were included. Doses and formulations were not recorded.

### Linear Analysis

A one-sample MR analysis was performed. Linkage disequilibrium between lipid-associated variants was addressed in the original source GWAS using study-specific approaches. Willer et al. defined distinct loci as being > 1 Mb apart and approximately independent (pairwise r^^2^ < 0.10)^17^. Hoffman et al. identified independent signals using stepwise conditional regression within 1-Mb windows centred on lead variants, with windows extended in regions of long-range LD^18^. Klarin et al. used conditional and joint analysis (GCTA-COJO) to identify independent genome-wide-significant lipid-associated variants [19]. Pairwise Pearson correlation coefficients (r) between the included genetic variants was calculated within UK Biobank and based on these all r² values were < 0.20. Linear regression models were used to quantify the association between genetic instruments and the exposure, with statin use included as a covariate. Cox proportional hazards models were used to quantify the association between genetic instruments and the outcome (SAH), with statin use again included as a covariate. Primary analyses were performed using inverse variance weighted method. Secondary analyses were performed using simple median and weighted median methods. MR-Egger method was performed to test for pleiotropic effects and provide an estimate of effect. MR-PRESSO method was performed to test and correct for pleiotropy.

### Nonlinear Analysis

For comparison, multivariable Cox proportional hazards models were used to characterise the nonlinear association between exposure (TC, HDL, LDL, TG) and outcome (SAH), with statin use included as a covariate. Hazard ratios (HR) were plotted as a function of lipid level (TC, HDL, LDL, TG) using models including natural cubic splines. Cubic spline interpolation fits cubic polynomials at specific intervals in the data to create curve fitting nonlinear data. For nonlinear MR, a piecewise-linear MR method was employed. For each participant, a weighted GRS was calculated by multiplying the number of effect alleles at each included SNP by its SNP-specific association with the corresponding lipid trait and summing these values across all included SNPs. Using the GRS, the expected value of each exposure at a GRS of 0 was estimated. Based on this value, participants were stratified into 10 strata based on the exposure distribution. The stratum-specific MR estimates were plotted with each slope representing the localised average causal effect (LACE) estimate for that stratum. Point-wise confidence intervals were calculated by re-sampling the MR estimates and re-calculating the full piecewise-linear shape. All analyses were performed in statistical software package R version 4.0.3 (packages including ‘survival’, ‘MendelianRandomization’ and ‘SUMnlmr’).

## Results

### Systematic Review

Overall, eight MR studies met the inclusion criteria [8–15]. Study characteristics are shown in Table 2. All studies performed two-sample analyses. All but one study used the GWAS published by Bakker et al. for the outcome cohort [20]. For the exposure cohort, 4 studies used the GWAS published by Willer et al.^10–13,17^. All studies reported on HDL, LDL and TG as exposures. Four studies included TC as an exposure. All but one study provided primary analyses combining RIA and UIA cases as an outcome. Five studies reported separately on RIA and four studies on UIA. No study provided data on statin use.

**Table 2:** Summary for studies included in the systematic review

Study	Date	Design	Exposure	Exposure cohort/ GWAS	Sample size	LD clumping	Instrument strength	Number of SNPs	Outcome	Outcome cohort/ GWAS	Controls	Cases	Results OR (CI)	Multiple comparison correction	Pleiotropy	Manuscript Conclusion
Karhunen et al. [8]	2021	Two sample	LDL	Meta- analysis (European, East Asian, South Asian and African)	188,577	r² < 0.01	Not reported	88	IA	Bakker et al.	59,544	6252	0.90 (0.78–1.02)	Bonferroni correction	Nil	Weak evidence for TG associated with IA/ RIA and LDL inversely associated with IA/ RIA
			HDL					113					0.93 (0.82–1.05)			
			TG					60					1.19 (1.02–1.38)			
			LDL	Meta- analysis (European, East Asian, South Asian and African)	188,577			88	RIA	Bakker et al.	59,544	4196	0.89 (0.77–1.02)			
			HDL					113					0.94 (0.80–1.09)			
			TG					60					1.14 (0.96–1.35)			
Chen et al. [9]	2021	Two sample	LDL	Meta- analysis (European, East Asian, South Asian and African)	188,577	r² < 0.001	F > 10	79	IA	Bakker et al. and UK Biobank	306,882	10,754	0.92 (0.81–1.04)	Bonferroni correction	Nil	Lipid dysfunction a causal risk factor for IA
			HDL					88					0.87 (0.75- 1.00)			
			TC					86					0.93 (0.84–1.04)			
			TG					55					1.23 (1.06–1.42)			
Zhang et al. [10]	2022	Two sample	LDL	UK Biobank (UK)	343,992	r² < 0.001	Not reported	116	IA	Bakker et al.	71,934	7495	0.86 (0.69–1.06)	Bonferroni correction	Nil	HDL and LDL reduce risk of UIA/ RIA
			HDL					279					0.82 (0.72–0.93)			
			TG					236					1.15 (1.00- 1.32)			
			LDL	UK Biobank (UK)	343,992			116	UIA	Bakker et al.	71,934	2070	0.91 (0.67–1.23)			
			HDL					278					1.02 (0.83–1.26)			
			TG					234					1.13 (0.92–1.39)			
			LDL	UK Biobank (UK)	343,992			116	RIA	Bakker et al.	71,934	5140	0.85 (0.67–1.08)			
			HDL					279					0.78 (0.66–0.91)			
			TG					236					1.17 (1.00- 1.38)			
Tian et al. [11]	2022	Two sample	LDL	Million Veteran Program (USA)	312,571	r² < 0.01	Not reported	127	IA	Bakker et al.	306,882	10,754	0.89 (0.78–1.02)	Bonferroni correction	Nil	Blood lipids suggestively associated with IA
			HDL					193					0.93 (0.83–1.03)			
			TC					139					0.86 (0.75–0.99)			
			TG					155					1.08 (0.97–1.20)			
Sun et al. [12]	2022	Two sample	LDL	Meta- analysis (European, East Asian, South Asian and African)	188,577	r² < 0.1	Not reported	44	IA	Bakker et al.	71,934	7495	1.10 (0.91–1.34)	Bonferroni correction	Nil	No conclusion
			HDL					59					1.08 (0.91–1.27)			
			TC					55					1.03 (0.85–1.24)			
			TG					28					0.81 (0.63–1.03)			
			LDL	Meta- analysis (European, East Asian, South Asian and African)	188,577			44	UIA	Bakker et al.	71,934	2070	1.22 (0.94–1.59)			
			HDL					59					1.08 (0.86–1.36)			
			TC					55					1.17 (0.89–1.53)			
			TG					28					1.06 (0.75–1.49)			
			LDL	Meta- analysis (European, East Asian, South Asian and African)	188,577			44	RIA	Bakker et al.	71,934	5140	1.06 (0.85–1.33)			
			HDL					59					1.06 (0.88–1.28)			
			TC					55					0.98 (0.78–1.23)			
			TG					28					0.71 (0.53–0.94)			
Acosta et al. [13]	2022	Two sample	LDL	Three GWAS (European, East Asian, South Asian and African)	596,002	r² < 0.1	Not reported	Not reported	IA	Bakker et al.	306,882	10,754	0.73 (0.57–0.94)	Not reported	Sig.	Inverse causal relationship between LDL and IA
			HDL										1.00 (0.61–1.65)			
			TG										1.23 (0.93–1.64)			
Li et al. [14]	2025	Two sample	LDL	UK Biobank (UK)	415,119	r² < 0.001	F > 10	160	UIA	FinnGen	412,105	3014	0.85 (0.71–1.02)	False Discovery Rate correction	Nil	No association after adjusting by multivariable analysis
			HDL					326					0.84 (0.73–0.96)			
			TG					293					1.09 (0.95–1.25)			
			LDL	UK Biobank (UK)	412,742			160	RIA	FinnGen	408,928	3814	0.89 (0.75–1.06)			
			HDL					326					0.88 (0.79–0.98)			
			TG					293					1.04 (0.91–1.18)			
Qiu et al. [15]	2025	Two sample	LDL	Global Lipids Genetics Consortium (European)	1,320,658	r² < 0.001	F > 10	204	IA	Bakker et al.	71,934	7495	0.95 (0.82–1.11)	Bonferroni correction	Nil	Genetically proxied TG and HDL associated with the risk of IA
			HDL					287					0.93 (0.88–0.98)			
			TC					234					0.92 (0.80–1.07)			
			TG					252					1.25 (1.07–1.47)			
			LDL	Global Lipids Genetics Consortium (European)	1,320,658			197	UIA	Bakker et al.	71,934	2070	0.98 (0.75–1.29)			
			HDL					300					0.93 (0.85–1.02)			
			TC					238					1.09 (0.84–1.40)			
			TG					250					1.33 (1.00- 1.77)			
			LDL	Global Lipids Genetics Consortium (European)	1,320,658			206	RIA	Bakker et al.	71,934	5140	0.99 (0.82–1.18)			
			HDL					294					0.95 (0.89–1.01)			
			TC					233					0.89 (0.75–1.06)			
			TG					250					1.23 (1.02–1.48)			

**Fig. 1:**
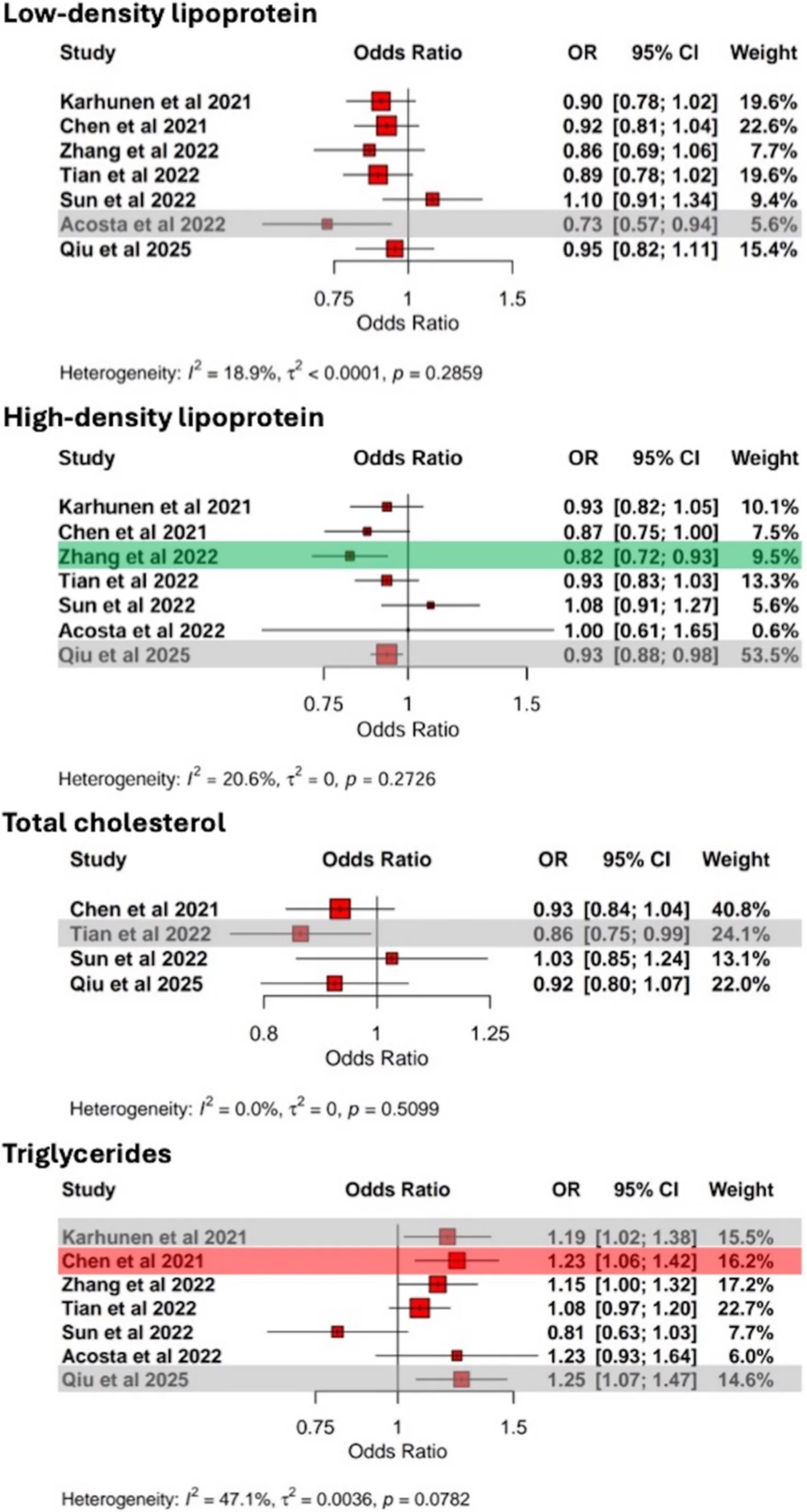
Forest plots for LDL cholesterol, HDL cholesterol, total cholesterol, and triglyceride. Green represents a significant inverse association. Red represents a significant association. Grey represents associations which were not significant following multiple-testing correction

A formal meta-analysis was not performed because most studies used the same or overlapping outcome data, meaning that the effect estimates were not independent. Therefore, the forest plots in Fig. 1 do not include summary statistics. Five studies reported a protective association for one lipid (LDL, HDL and TC) [10, 11, 13–15]. Three studies reported a protective association for HDL, however, in two studies this effect was not significant after correcting for multiple analyses or performing multivariable adjustment [10, 14, 15]. The direction of the effect was in the opposite direction of what might have been anticipated for LDL [13]. Three studies found an association between SAH and TG. However, after correction for multiple testing and multivariable adjustment this effect was not present in two of these studies [8, 9, 15]. Despite using similar cohorts there was a degree of heterogeneity between studies which almost reached significance (Fig. 1). In subgroup analyses of RIA and UIA, there were also several isolated significant findings with a similar level of heterogeneity present (Table 2). One study did not account for multiple comparisons and found significant evidence of pleiotropy [13]. The remaining studies did correct for multiple analyses. Overall, only two of six positive findings were shown to be significant after correction for multiple comparisons (one HDL and one TG) [9, 10].

### Linear Mendelian Randomisation Analysis

**Fig. 2:**
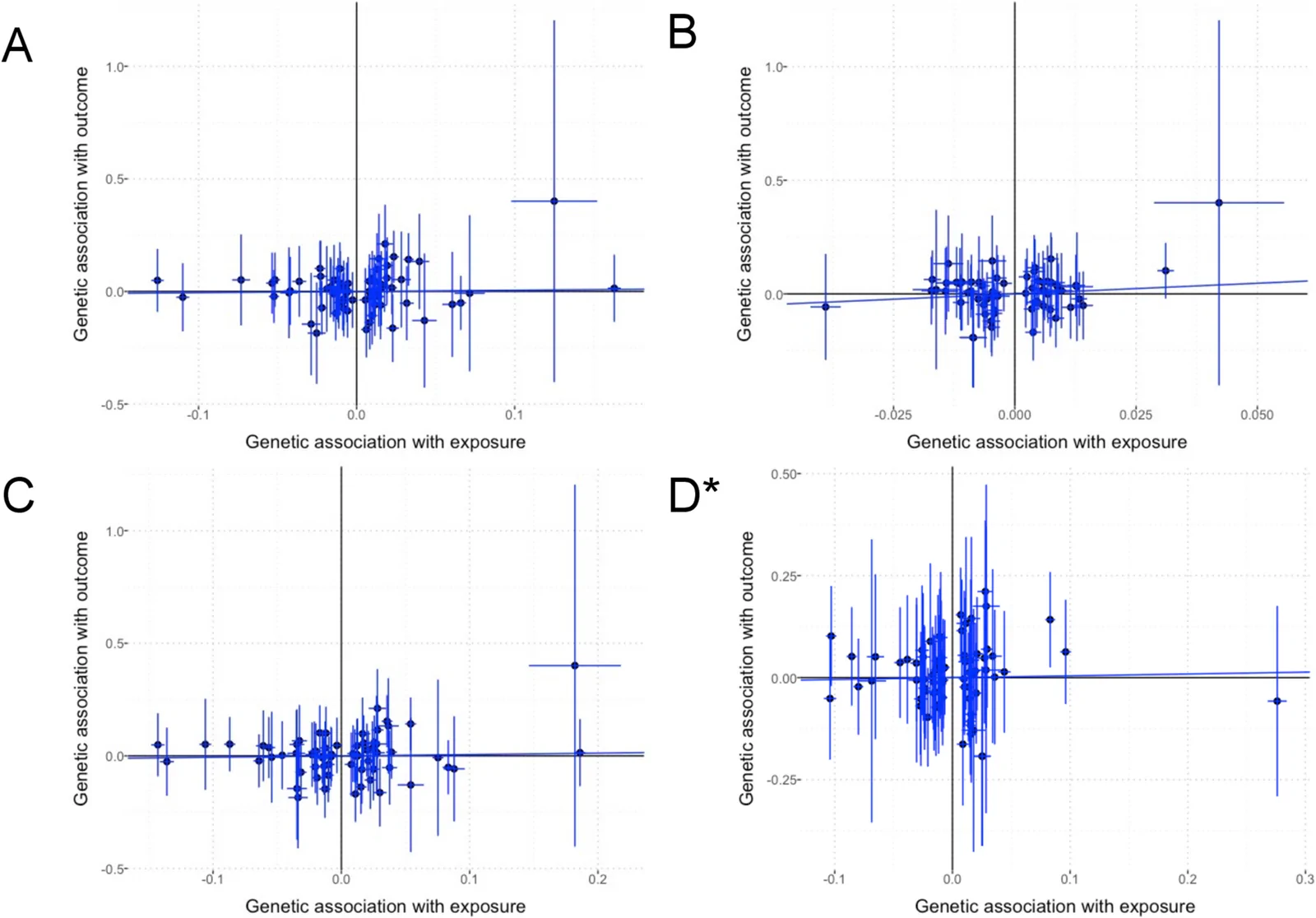
MR plots for LDL cholesterol (**A**), HDL cholesterol (**B**), total cholesterol (**C**) and triglyceride (**D**). Each plot shows the effect estimates of association tests between the SNPs and both the exposure and outcome. The blue summary line corresponds to the inverse variance weighted method slope. *a single SNP was excluded to improve the scale of the figure

A total of 486,603 eligible participants from the UK Biobank were included in this analysis. There were a total of 560 SAH cases on follow up with a mean follow up length of 8.05 years. A total of 117 SNPs were screened for their association with lipids. Overall, 106 SNPs were included in the analyses, 70 for LDL, 63 for HDL, 67 for TC and 74 for TG. MR plots for the primary analysis are shown in Fig. 2. Inverse variance weighted method found no significant association between SAH and LDL (HR 1.06 [0.64–1.73]), HDL (HR 2.57 [0.42–15.74]), TC (HR 1.06 [0.71–1.60]), or TG (HR 1.05 [0.69–1.59]). Similar results were seen for simple median, weighted median and MR-Egger methods (Table 3). The MR-Egger method found no significant evidence of directional pleiotropy in the primary analysis (*p* > 0.05). MR-PRESSO method found no horizontal pleiotropic outlier variants in the primary analysis.

**Table 3:** Results of the linear MR analysis

Lipid trait	SNP threshold	Method	SAH				aSAH			
			HR	CI		*P*-value	HR	CI		*P*-value
LDL	**F statistic > 10**	**Simple median**	1.12	0.40	3.15	0.83	1.38	0.47	4.03	0.56
		**Weighted median**	1.03	0.54	1.98	0.93	0.94	0.48	1.83	0.86
		**IVW**	1.06	0.64	1.73	0.83	1.03	0.63	1.67	0.91
		**MR-Egger**	0.91	0.48	1.76	0.79	0.85	0.45	1.61	0.62
	**F statistic > 20**	**Simple median**	1.26	0.46	3.48	0.65	1.02	0.36	2.92	0.97
		**Weighted median**	1.00	0.52	1.89	0.99	0.94	0.48	1.83	0.86
		**IVW**	1.07	0.66	1.76	0.77	1.03	0.64	1.69	0.89
		**MR-Egger**	0.76	0.38	1.49	0.42	0.73	0.37	1.44	0.37
	**F statistic > 50**	**Simple median**	1.09	0.46	2.59	0.84	0.95	0.39	2.31	0.91
		**Weighted median**	0.99	0.51	1.93	0.98	0.94	0.49	1.81	0.85
		**IVW**	1.01	0.60	1.70	0.97	0.96	0.58	1.58	0.86
		**MR-Egger**	0.68	0.30	1.53	0.36	0.70	0.32	1.55	0.38
HDL	**F statistic > 10**	**Simple median**	4.37	0.20	96.16	0.35	1.62	0.06	41.43	0.77
		**Weighted median**	4.48	0.25	80.64	0.31	2.33	0.12	46.25	0.58
		**IVW**	2.57	0.42	15.74	0.31	2.01	0.31	13.14	0.47
		**MR-Egger**	1.30	0.05	32.66	0.87	1.59	0.06	43.90	0.78
	**F statistic > 20**	**Simple median**	5.12	0.28	94.73	0.27	1.62	0.08	32.85	0.75
		**Weighted median**	4.51	0.27	76.25	0.30	2.47	0.14	43.12	0.54
		**IVW**	2.87	0.46	17.94	0.26	2.17	0.32	14.45	0.42
		**MR-Egger**	0.77	0.03	23.13	0.88	1.22	0.04	41.72	0.91
	**F statistic > 50**	**Simple median**	0.45	0.02	8.95	0.60	0.73	0.03	16.78	0.84
		**Weighted median**	4.35	0.28	67.02	0.29	1.96	0.11	34.33	0.64
		**IVW**	1.21	0.17	8.40	0.85	0.95	0.13	7.13	0.96
		**MR-Egger**	4.12	0.06	268.54	0.51	10.50	0.14	803.52	0.29
TC	**F statistic > 10**	**Simple median**	1.11	0.53	2.32	0.77	1.02	0.47	2.19	0.96
		**Weighted median**	1.02	0.59	1.75	0.95	0.94	0.54	1.64	0.83
		**IVW**	1.06	0.71	1.60	0.76	1.02	0.68	1.52	0.93
		**MR-Egger**	0.96	0.54	1.69	0.88	0.90	0.52	1.57	0.72
	**F statistic > 20**	**Simple median**	1.12	0.52	2.39	0.78	1.02	0.46	2.24	0.97
		**Weighted median**	0.98	0.57	1.67	0.94	0.94	0.55	1.62	0.83
		**IVW**	1.10	0.73	1.65	0.66	1.05	0.70	1.56	0.82
		**MR-Egger**	0.81	0.45	1.47	0.49	0.77	0.44	1.37	0.38
	**F statistic > 50**	**Simple median**	0.76	0.41	1.40	0.38	0.75	0.39	1.43	0.39
		**Weighted median**	0.96	0.57	1.63	0.89	0.94	0.54	1.63	0.82
		**IVW**	0.99	0.66	1.48	0.97	0.93	0.62	1.38	0.71
		**MR-Egger**	0.77	0.40	1.48	0.43	0.78	0.40	1.49	0.45
TG	**F statistic > 10**	**Simple median**	1.20	0.46	3.09	0.71	0.98	0.37	2.61	0.97
		**Weighted median**	0.95	0.51	1.80	0.88	0.79	0.41	1.53	0.48
		**IVW**	1.05	0.69	1.59	0.83	1.01	0.66	1.54	0.97
		**MR-Egger**	1.04	0.59	1.83	0.89	1.00	0.56	1.75	0.99
	**F statistic > 20**	**Simple median**	1.15	0.49	2.69	0.75	0.93	0.38	2.25	0.87
		**Weighted median**	0.95	0.50	1.81	0.88	0.79	0.41	1.52	0.48
		**IVW**	1.02	0.68	1.54	0.92	0.99	0.65	1.51	0.96
		**MR-Egger**	1.17	0.66	2.06	0.60	1.08	0.60	1.94	0.81
	**F statistic > 50**	**Simple median**	1.38	0.57	3.32	0.47	1.08	0.42	2.75	0.87
		**Weighted median**	0.96	0.51	1.80	0.90	0.82	0.43	1.57	0.55
		**IVW**	1.11	0.73	1.69	0.63	1.06	0.69	1.64	0.79
		**MR-Egger**	0.95	0.51	1.78	0.88	0.90	0.47	1.73	0.76

As a sensitivity analysis, additional models were fitted adjusting for statin use and other lipid-lowering therapies, including cholesterol absorption inhibitors, PCSK9 inhibitors, bile acid sequestrants, fibrates, niacin and omega-3 fatty acids. These analyses did not find any significant association for LDL (IVW HR 1.08 [0.66–1.77]), HDL (IVW HR 2.46 [0.40- 15.23]) and TC (IVW HR 1.08 [0.72–1.62]). Further analyses were performed using SNPs more strongly associated with exposure (F > 20 and F > 50, Table 3). For F > 20, no effect was found for LDL (IVW HR 1.07 [0.66–1.76]), HDL (IVW HR 2.87 [0.46–17.94]), TC (IVW HR 1.10 [0.73–1.65]), and TG (IVW HR 1.02 [0.68–1.54]). For F > 50, a similar effect was found for LDL (IVW HR 1.01 [0.60–1.70]), HDL (IVW HR 1.21 [0.17–8.40]), TC (IVW HR 0.99 [0.66–1.48]), and TG (IVW HR 1.11 [0.73–1.69]). Analyses performed investigating cases with ICD codes specifying an aneurysmal SAH also did not influence the results (Table 3).

### Nonlinear Analysis

**Fig. 3:**
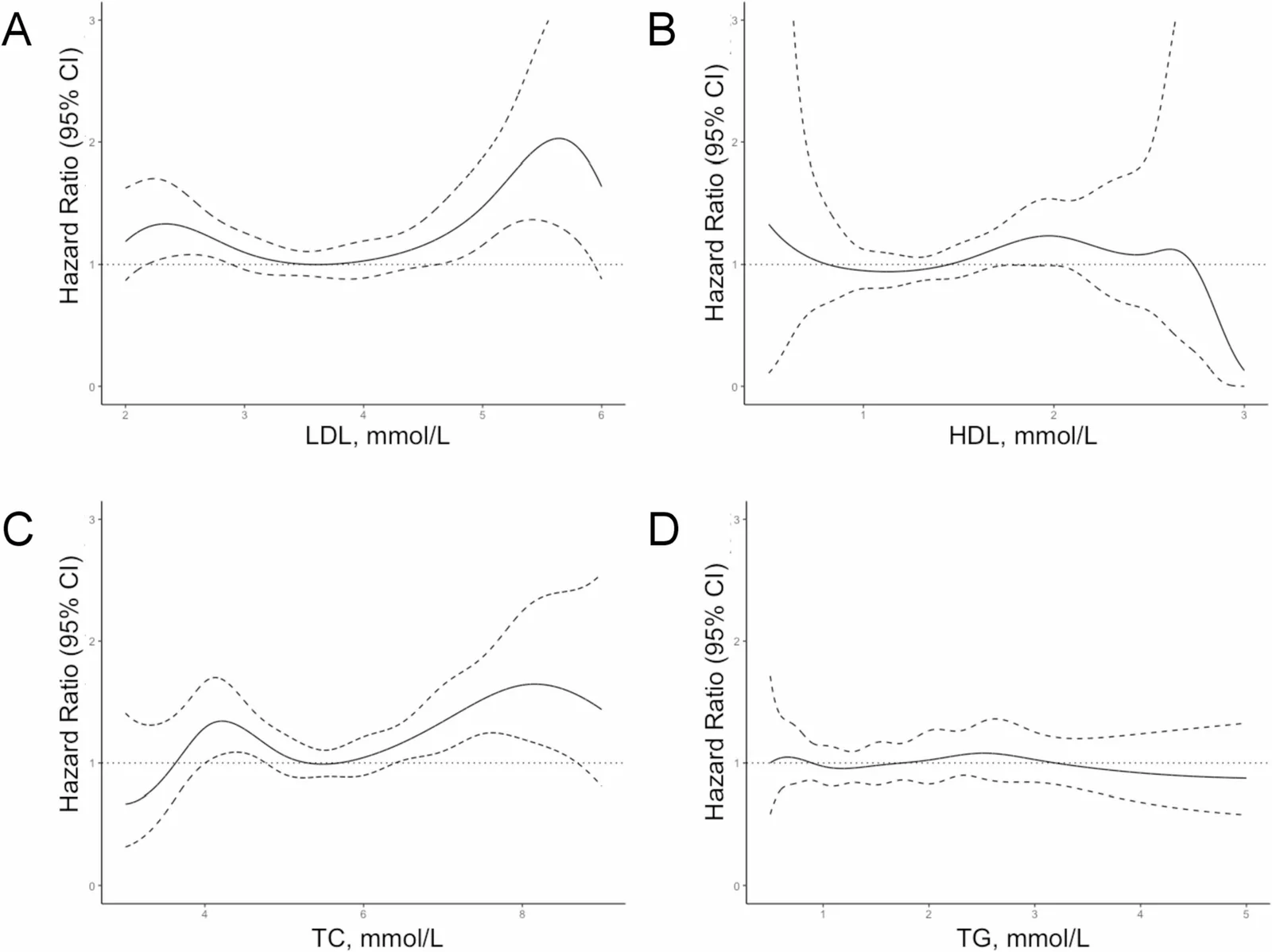
Non-linear models of observational data including natural cubic splines. Solid line represents the hazard ratio and dotted line the 95% confidence interval. Reference values; **A** 3.56, **B** 1.45, **C** 5.69, **D** 1.75

**Fig. 4:**
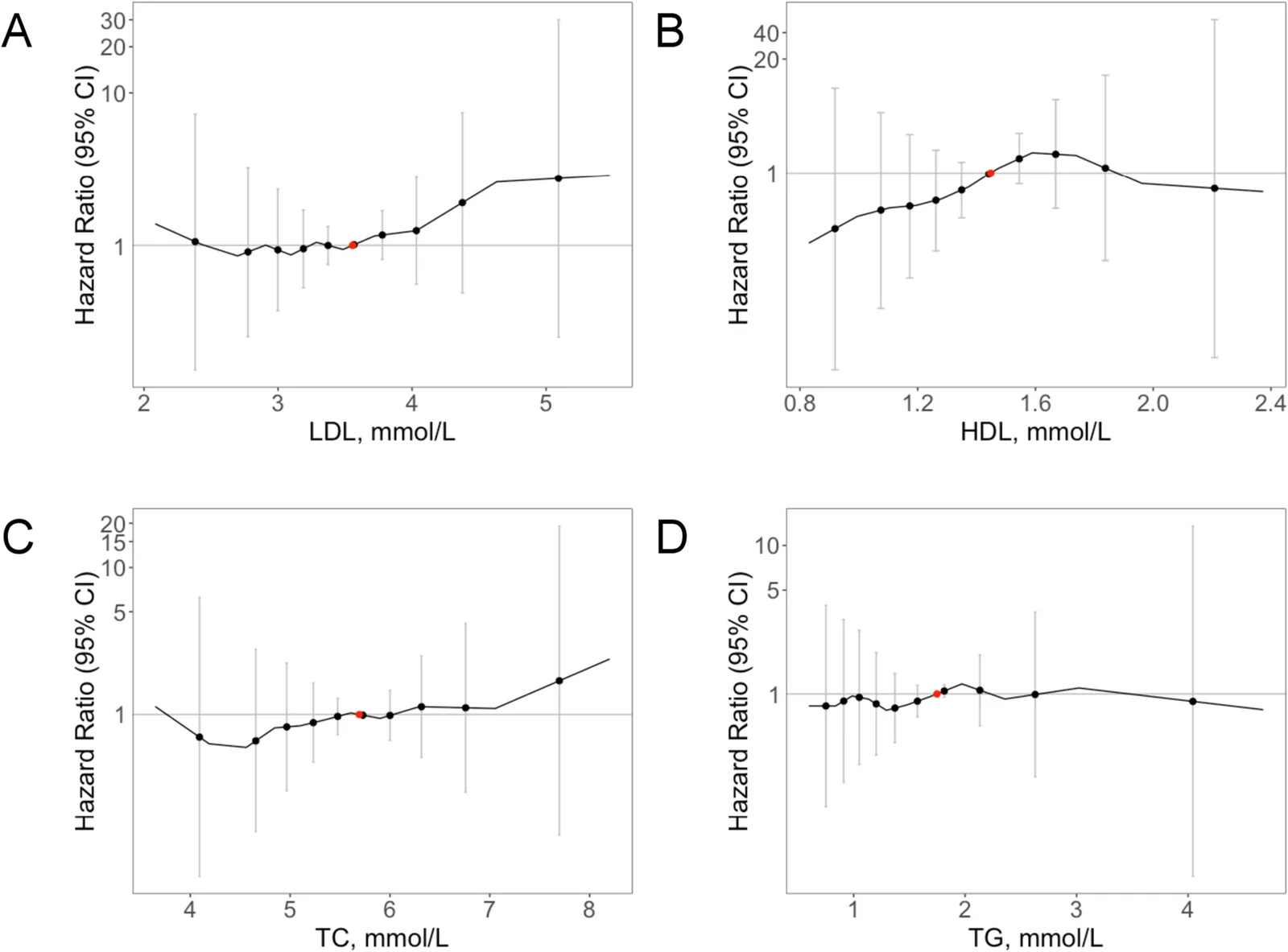
Non-linear MR models plotted as a piecewise-linear function, where the slope in each piece is the LACE estimate in that stratum. Point-wise 95% confidence intervals. Reference values; **A** 3.56, **B** 1.45, **C** 5.69, **D** 1.75

Linear Cox proportional hazards models accounting for statin use found no significant association between SAH and LDL (HR 1.06 [0.96–1.18]), HDL (HDL 1.18 [0.93–1.48]), TC (HR 1.07 [0.99–1.16]) or TG (HR 1.00 [0.92–1.08]). Nonlinear analyses including cubic splines are shown in Fig. 3. Apart from TG, the remaining lipid markers showed visual evidence suggestive of nonlinearity in the spline models. In particular, LDL and TC demonstrated curves which could be interpreted as U-shaped. However, these patterns were inconsistent, and statistical tests of nonlinearity were not significant. The results of the nonlinear MR analysis are shown in Fig. 4. The curves in Fig. 4 have a visual resemblance to those in Fig. 3. However, again statistical tests of nonlinearity were not significant (LDL: Quadratic p value = 0.25, Cochran p value = 0.52; HDL: Quadratic p value = 0.14, Cochran p value = 0.79; TC: Quadratic p value = 0.28, Cochran p value = 0.70). Subsequently, these observations should be interpreted cautiously and not considered evidence of nonlinear effects.

## Discussion

In this study, we have investigated the genetic association between lipid traits and SAH by performing a systematic review and MR analysis of the UK Biobank to address shortcomings in the existing literature. The systematic review identified eight MR studies, which were largely based on the same outcome cohort but remarkably all had different findings. Given the promise that MR would provide a robust assessment of causal relationships between exposures it is unexpected to see such heterogenous results. This is especially the case when MR should be particularly suited to lipid traits, where the genetic instruments are well defined. The exact reason for this heterogeneity remains unclear.

All MR studies are reliant on the assumption that (1) genetic instruments are strongly associated with the exposure, (2) genetic instruments are not associated with confounders and (3) genetic instruments only influence the outcome via the exposure. The assumption that lipid traits make a good candidate for MR given their strong association with genetic instruments may not hold. There is potential evidence for this given that several studies used the same exposure cohort but included different numbers of SNPs due to differing thresholds used to account for weak instrument bias and linkage disequilibrium. It is possible that this may represent a limitation of the studies and explain the heterogeneous findings.

The SNP–exposure and SNP-outcome associations may also be influenced by confounding factors. Only a minority of previous studies performed multivariable Mendelian randomisation analyses. These were primarily used to estimate the direct effects of correlated lipid traits [9, 10, 13], whereas only one study examined components of the metabolic syndrome [14]. The most obvious confounder would be lipid-lowering medications, such as statins, which are more likely to be used in patients with a genetic predisposition to higher circulating lipid levels. Importantly, statins may also influence the association between genetic instruments and SAH through other routes. Unlike previous MR studies where presumably there was insufficient data, our analysis included sensitivity analyses adjusting for statin use and other lipid-lowering therapies. Although this represents an important methodological advantage, adjustment for statin use cannot fully account for medication-related bias, and residual confounding or collider bias cannot be excluded.

In addition to the general assumptions of MR that may have been violated, the available studies were all two-sample analyses that come with further assumptions. It is assumed that (1) both samples represent the same underlying population and (2) there is no overlap in participants between the two samples. However, the studies used originated from different populations, with clear examples where the two cohorts did not represent the same underlying population (UK, American and Finnish) (Table 2) [11, 14]. Additionally, there was an overlap of participants in several studies with three groups of patients included in both exposure and outcome cohorts (Northern Finland Birth Cohort 1966, HUNT study, and UK Biobank) (Table 2) [8–10, 12, 13, 15]. In fact, a single study combined two outcome cohorts in a single analysis both of which included data from the UK Biobank (Table 2) [9]. The presence of this overlap can lead to bias and a higher risk of type I errors. Therefore, the validity of two-sample MR depends on the quality of the underlying studies, which in this case used a case–control design with cases and controls drawn from different populations.

Lastly, two-sample MR analyses rely on summary-level data and therefore assume a linear relationship between the exposure and outcome. It is plausible that the relationship between lipids and SAH is nonlinear. For example, both high and low lipid levels could be associated with increased risk, producing a U-shaped relationship. Fitting a linear model to this would yield unstable results and could contribute to the variation between studies.

We concluded that the existing literature does not demonstrate a consistent association between lipid traits and SAH, but a true genetic association could have been missed either because (a) no account was taken of lipid-lowering medications which are both in common use and influence lipid levels, or (b) the relationship between some lipids and SAH are not linear. We aimed to test this using a one-sample MR analysis that permits assessment of nonlinear effects while accounting for statin use.

We did not find any evidence of a relationship between any lipid trait and SAH. This finding was consistent across all MR methods and sensitivity analyses, with no suggestion of any underlying trend in the estimates or plots to indicate a clinically meaningful relationship. Although there were occasional visual suggestions of nonlinearity, these were inconsistent and not statistically supported. Nevertheless, modest effects cannot be excluded, particularly given the limited number of outcome events and the inherent limitations of one-sample MR.

The main limitation of our MR analysis is the relatively small number of SAH events. Despite including half a million participants, these analyses have limited statistical power to detect modest effects, particularly for HDL where confidence intervals are wide. This is particularly true for the nonlinear MR analyses as the number of SAH events was further divided across strata, reducing statistical power. However, while modest effects and subtle nonlinear effects cannot be excluded, it was notable that all analyses were consistently negative, and there was little suggestion of an association that might have reached significance with larger numbers.

Although one-sample MR offers several advantages over two-sample MR, including the ability to adjust for statin use and investigate nonlinear associations, there remain important limitations. Weak instrument bias, residual confounding, and population stratification may influence estimates. Furthermore, given that UK Biobank participants are healthier than the general population, selection bias that may limit the generalisability of the findings.

A further limitation is the identification of SAH using linked hospital admission and death registry ICD-10 codes rather than imaging-confirmed or clinically adjudicated diagnoses. Although traumatic haemorrhages were excluded and sensitivity analyses restricted to aneurysmal SAH produced similar findings, some outcome misclassification remains possible and would be expected to bias effect estimates towards the null.

While we did not perform multivariable MR incorporating established aneurysm risk factors such as smoking and hypertension, MR is less susceptible to confounding and reverse causation, and lipid traits are supported by strong genetic instruments. However, future multivariable analyses may help clarify whether lipid traits exert independent effects beyond established aneurysm risk factors.

Taken together, the available evidence does not support a major relationship between lipid traits and SAH. The inconsistency of previous MR studies, combined with the absence of an association in the present analysis, suggests that reported associations may reflect methodological differences or unstable estimates rather than a consistent biological effect. However, small effects cannot be excluded, particularly given the limited number of SAH events in this cohort, and these results should not be interpreted as evidence against potential lipid-independent effects of statins or other lipid-lowering therapies on aneurysm formation or rupture.

## Supplementary Information

Below is the link to the electronic supplementary material.

Supplementary Material 1 (XLSX 60.4 KB)

Supplementary Material 2 (JPG 331 KB)

## Data Availability

All data are available from the UK Biobank. Informed consent was obtained from the participants.
